# Environmental suitability for *Lutzomyia* (*Nyssomyia*) *whitmani* (Diptera: Psychodidae: Phlebotominae) and the occurrence of American cutaneous leishmaniasis in Brazil

**DOI:** 10.1186/s13071-018-2742-7

**Published:** 2018-03-07

**Authors:** Simone Miranda da Costa, José Luís Passos Cordeiro, Elizabeth Ferreira Rangel

**Affiliations:** 1 Laboratório Interdisciplinar de Vigilância Entomológica em Diptera e Hemiptera, Instituto Oswaldo Cruz, FIOCRUZ, Rio de Janeiro, Brasil; 2 Fiocruz da Mata Atlântica, Fundação Oswaldo Cruz, FIOCRUZ, Rio de Janeiro, Brasil

**Keywords:** Climate change, Ecological niche modelling, *Lutzomyia* (*N*.) *whitmani*, Cutaneous leishmaniasis

## Abstract

**Background:**

Leishmaniasis represents an important public health problem in Brazil. The continuous process of urbanization and expansion of human activities in forest areas impacts natural habitats, modifying the ecology of some species of *Leishmania*, as well as its vectors and reservoirs and, consequently, changes the epidemiological pattern that contributes to the expansion of American cutaneous leishmaniasis in Brazil. Here, we discuss *Lutzomyia* (*Nyssomyia*) *whitmani*, the main vector of ACL, transmitting two dermotropic *Leishmania* species including *Leishmania* (*Viannia*) *braziliensis* and *Leishmania* (*V*.) *shawi*.

**Methods:**

We used the maximum entropy niche modelling approach (MaxEnt) to evaluate the environmental suitability of *L.* (*N*.) *whitmani* and the transmission of ACL in Brazil, in addition to designing models for a future scenario of climate change. MaxEnt was used under the “auto-features” mode and the default settings, with 100-fold repetition (bootstrap). The logistic output was used with higher values in the habitat suitability map, representing more favourable conditions for the occurrence of *L.* (*N*.) *whitmani* and human cases of ACL.

**Results:**

Two models were developed: the *Lutzomyia* (*N.*) *whitmani* model (LWM) and the American cutaneous leishmaniasis model (ACLM). LWM identified the species “preferential habitat” included regions with moderate annual precipitation (AP) between 1000–1600 mm, intermediate vegetation density (NDVI) values, mean temperature of the coldest quarter (MTCQ), between 15–21 °C, and annual mean temperature (AMT), between 19–24 °C. ACLM indicates that ACL is strongly associated with areas of intermediate density vegetation, areas with AP between 800–1200 mm, MTCQ above 16 °C and AMT below 23 °C.

**Conclusions:**

The models generated for *L.* (*N.*) *whitmani* and ACL indicated a satisfactory predictive capacity. Future projections of LWM indicate an expansion of climatic suitability for *L.* (*N*.) *whitmani* for the northern and southern regions of Brazil. Future projections of ACL indicate the ongoing process of disease expansion in the face of the predicted climatic changes and reinforce the broad geographical expanse of this disease in Brazil. The models were able to identify that a continuous process of environmental degradation favours the establishment of *L.* (*N*.) *whitmani* and the occurrence of ACL by a strong association of the vector(s) and ACL to areas of intermediate vegetation cover density.

**Electronic supplementary material:**

The online version of this article (10.1186/s13071-018-2742-7) contains supplementary material, which is available to authorized users.

## Background

The simplification of biological communities, the fragmentation and loss of habitats resulting from human occupation modify the parasite/host interactions, which may lead to the emergence and reemergence of several diseases in animal and human populations [1].

In the last decade, a growing number of studies have investigated the effects of biodiversity on the risk of disease occurrence, mainly due to the interest in identifying and evaluating the importance of biodiversity and the environmental services it provides [2]. The influence of diversity on transmission cycles has been described for some diseases [3, 4]. However, little is known about the ecological mechanisms related to these effects [5]. Understanding the structure and functioning of the ecological processes involved in the dynamics of the interactions between parasites, hosts, and the environment becomes critical to comprehend the relationship between biodiversity and the emergence or reemergence of zoonoses.

Due to new and complex epidemiological scenarios, leishmaniasis is considered a reemerging disease [6] modelling important public health problems in Brazil. American cutaneous leishmaniasis (ACL) represents an example of zoonosis related to land use and biodiversity management, both by the severity of the disease and by the direct relationship of elements and the environmental context (landscape) in its transmission cycle [7].

The circulation of phlebotomine sand fly vectors in environments outside the geographical limits of natural foci is increasing and leads to modifications in the classic epidemiological patterns of leishmaniasis. Such modifications are related to changes in the determinant factors for the exposure of man to transmission, demographic expansion and the process of urbanization on the limits of natural foci, as well as the occurrence of forest remnants adjacent to urban areas [6, 8, 9].

In this context, we highlight *L.* (*N*.) *whitmani*, a sand fly species registered in 26 of 27 Brazilian federative units [10] and a transmitter of two dermotropic leishmaniasis species including *Leishmania* (*V*.) *shawi* in the Amazon, and *Leishmania* (*V*.) *braziliensis* in the North, Northeast, Midwest, Southeast and South Regions [11, 12]. The former presents different behaviour in different regions, has a wide geographical distribution, and is adapted to several climates and types of vegetation cover [10, 12, 13]. This ecological plasticity reflects the occurrence of this species in all epidemiological patterns described for ACL [9]. Throughout the Brazilian territory, according to qualitative changes related to antrophilia and domesticity, Lainson [14] suggested that *L.* (*N*.) *whitmani* represented a complex of cryptic species.

The characterization of factors influencing the spatial distribution of *L.* (*N*.) *whitmani*, in general, has been an efficient tool for a better understanding of ecological processes. The ecological niche models (ENM) has been widely used as a tool to describe environmental conditioning factors and to identify patterns related to environmental suitability for species occurrence [15, 16]. In recent years, many techniques for modelling niches and species distributions have been developed and applied extensively in biogeography, ecology and conservation studies [15, 17, 18]. The maximum entropy model (MaxEnt) [19] is consistently competitive with the highest performing methods and is one of the most common approaches used to determine geographical distribution and ecological features of species [20, 21].

Peterson & Shaw [22] modelled three sand fly vector species from South America, *L.* (*N*.) *whitmani*, *Lutzomyia* (*Nyssomyia*) *intermedia* and *Lutzomyia migonei*, and identified an increase in areas of climate suitability for the year 2050. According to the models, *L.* (*N*.) *whitmani* presented the greatest areas of dispersion. The purpose of the present study was to evaluate the environmental suitability and project future scenarios (*via* ENM), for *L.* (*N*.) *whitmani* and ACL in Brazil, in the face of global climate change.

## Methods

### Occurrence data

For data related to the occurrence of the disease, we used municipalities with records of endemic areas for ACL, based on the ACL Data Banks, for the period 2003 to 2013, provided by the Brazilian Ministry of Health (*n* = 1882, of which 1506 were used for modeling and 376 for additional accuracy test) (see Additional file 1: Table S1). For *L.* (*N*.) *whitmani* occurrence, the municipalities with a confirmed record of the vector (*n* = 992, of which 794 were used for modelling and 198 for additional accuracy test), were considered in the *L.* (*N*.) *whitmani* model (see Additional file 2: Table S2).

Occurrence data for *L.* (*N*.) *whitmani* was extracted from previously published data (until 2013) available from online databases including PubMed (http://www.ncbi.nlm.nih.gov/pubmed), the ISI Web of Knowledge (http://apps.webofknowledge.com), SCOPUS (http://www.scopus.com), and CAPES (http://catalogodeteses.capes.gov.br). We also collected unpublished records from the Health Department of Brazil and major Brazilian sand fly collections at the Centro de Pesquisas Rene Rachou, FIOCRUZ, the Instituto Evandro Chagas, IEC, and the Faculdade de Saude Publica, USP.

### Environmental descriptors

Ten environmental variables (0.04 ° of spatial resolution, ~5 km) were used, 8 of which were WorldClim [23] climatic variables, as well as data on altitude and vegetation indices, which are all displayed in Table 1.

**Table 1:** Environmental variables used to model the potential distribution of *Lutzomya* (*N*.) *whitmani* and American cutaneous leishmaniasis in Brazil. All variables were resampled from original resolution to 0.04° (~5 km), using the average value of all involved pixels, where the source pixels are covered by the target pixel

Environmental variable	Acronym	WorldClim Acronym	Source
Annual mean temperature	AMT	BIO1	WorldClim [23]
Mean temperature of wettest quarter	MTWEQ	BIO8	
Mean temperature of driest quarter	MTDQ	BIO9	
Mean temperature of warmest quarter	MTWAQ	BIO10	
Mean temperature of coldest quarter	MTCQ	BIO11	
Annual precipitation	AP	BIO12	
Precipitation of wettest quarter	PWQ	BIO16	
Precipitation of driest quarter	PDQ	BIO17	
Altitude - digital elevation model	ALT	–	Shuttle Radar Topography Mission (http://www2.jpl.nasa.gov/srtm/)
MODIS normalized difference vegetation index-32 day composites-Oct/15 - Nov/15/2004. Date of the composite represents well the contrast between forest and open formations.	NDVI	–	Global land cover facility (http://www.landcover.org/data/modis/)

To project future environmental conditions (i.e. 2050), we used two representative concentration pathways (RCPs) of the HadGEM2-ESgeneral circulation model: RCP 4.5 and RCP 8.5 greenhouse gas concentration trajectories adopted by Intergovernmental Panel on Climate Change (IPCC) for its fifth Assessment Report (AR5) in 2014 [24]. These were selected to represent contrasting scenarios in projections for climate change. RCP 4.5 represents a relatively optimistic scenario and assumes that the radiative forcing of greenhouse gas stabilizes shortly after 2100, and RCP 8.5, more pessimistic, radiative forcing keeps rising after 2100.

### Ecological niche models

We used the maximum entropy niche modelling approach, as implemented in MaxEnt software (version 3.3.3k), to evaluate the environmental suitability for *L.* (*N*.) *whitmani* and occurrence of ACL in Brazil, as well as to model projections for future climate change scenarios. The method considers the occurrence of *L.* (*N*.) *whitmani* in association with environmental variables [25], producing response curves that indicate how each variable affects the predicted distribution [26]. MaxEnt has been shown to be robust for ENM construction from presence-only data [24] and to describe the ecological and spatial relationships between species and environmental conditions.

MaxEnt was applied under the ‘auto-features’ mode, and the default settings, with 100-fold replicates generated by bootstrap [26]. The logistic output was used (habitat suitability on a scale of 0–1), with higher values in the habitat suitability map (HSM) representing more favourable conditions for the occurrence of *L.* (*N*.) *whitmani* or ACL. Two models were developed: (i) the *Lutzomyia* (*N*.) *whitmani* model (LWM), and (ii) American cutaneous leishmaniasis model (ACLM). Both models were developed using ten environmental variables, 80% of occurrence data for training and 20% for the test.

To infer the effect of climate change on the distribution of *L.* (*N*.) *whitmani* and ACL, each model was projected using the scenarios RCP 4.5 and RCP 8.5. For these projections, NDVI was removed, since this environmental variable had no projection for the future scenarios we used.

We assessed the accuracy of each model using AUC (area under the receiver operating characteristic [ROC] curve) on MaxEnt. Additionally, we used an independent set of 127 and 376 actual occurrence records, for *L.* (*N*.) *whitmani* and ACL human cases, respectively (randomly selected from total points and not used in the generation of models), to evaluate the predictive capacity of the models. The predicted suitability of the models was extracted for each test point using the ArcGIS 10.1 software (ESRI©), and the average suitability was used to evaluate model accuracy.

For *L.* (*N*.) *whitmani* and ACL, potential distribution binary maps (suitable/unsuitable) were applied the minimum training presence (MTP) as a threshold value for models, because it is the most conservative threshold, identifying the minimum predicted area possible while still maintaining a zero omission rate for both training and test data.

For comparative purposes, the images resulting from each model (with continuous values from 0 to 1) were reclassified into five environmental suitability zones: (i) unsuitable zone (UNSZ; value pixel suitability < minimum training presence, MTP); (ii) low suitability zone (LSZ, value pixel suitability between MTP value and 0.25); (iii) intermediate suitability zone (ISZ, value pixel suitability between 0.25 and 0.50); (iv) high suitability zone (HSZ, value pixel suitability 0.50 and 0.75); and (v) a very high suitability zone (VHSZ, value pixel suitability > 0.75).

### Model comparison

The ACLM and LWM were compared using Fuzzy for continuous maps, and the Kappa index for categorical maps (suitable/unsuitable) using the Map Comparison Kit v.3.2 software developed by the Netherlands Environmental Assessment Agency [27, 28]. Both indices express the pixel similarity for a value between 0 (fully distinct) and 1 (fully identical).

Additionally, we used Olson et al.’s [29] delineation of the terrestrial “Ecoregions of the World” and the Brazilian biomes [30] as a base map to better demonstrate the comparison between generated.

## Results

**Fig. 1:**
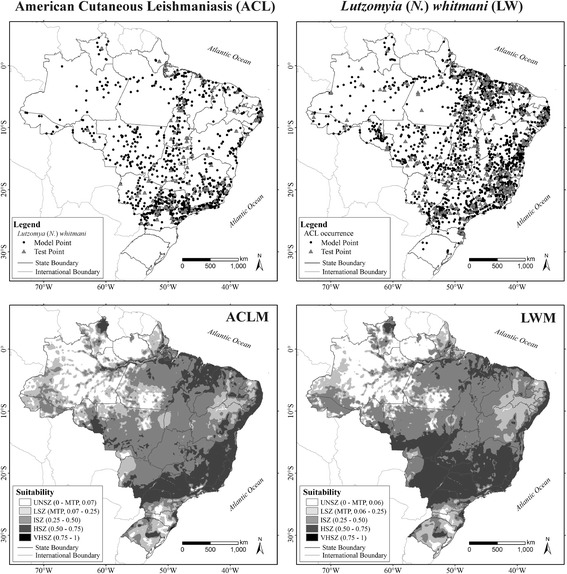
Occurrence data for American cutaneous leishmaniasis (ACL) and *Lutzomyia* (*N*.) *whitmani* (LW), and environmental suitability for ACL and LW in Brazil, current conditions. Unsuitable zone (UNSZ; value pixel suitability < minimum training presence, MTP), low suitability zone (LSZ, value pixel suitability between MTP value and 0.25), intermediate suitability zone (ISZ, value pixel suitability between 0.25–0.50), high suitability zone (HSZ, value pixel suitability between 0.50–0.75), and a very high suitability zone (VHSZ, value pixel suitability > 0.75) identified

With an average AUC of 0.77 (SD = 0.004; 100-fold replicates), ACLM achieved a satisfactory model fit, and the modelled distribution performed better than random. The predictive capacity of ACLM, evaluated by the average suitability test of 0.53 (SD = 0.12) in each test point, indicates that the model achieved high accuracy. This average value corresponds to the high suitability zone for ACL. Based on the minimum training presence (MTP = 0.07) cut off criteria (MTP = 0.07), ACLM identified many of the regions of Brazil appropriate for the occurrence of ACLM (Fig. 1), covering 82.3% of the Brazilian territory. LWM showed similar performance, with a mean AUC of 0.82 (SD = 0.006; 100-fold replicates) and average suitability test of 0.54 (SD = 0.15), indicating the satisfactory predictive capacity of both models (Fig. 1), covering 83.4% of the Brazilian territory.

**Fig. 2:**
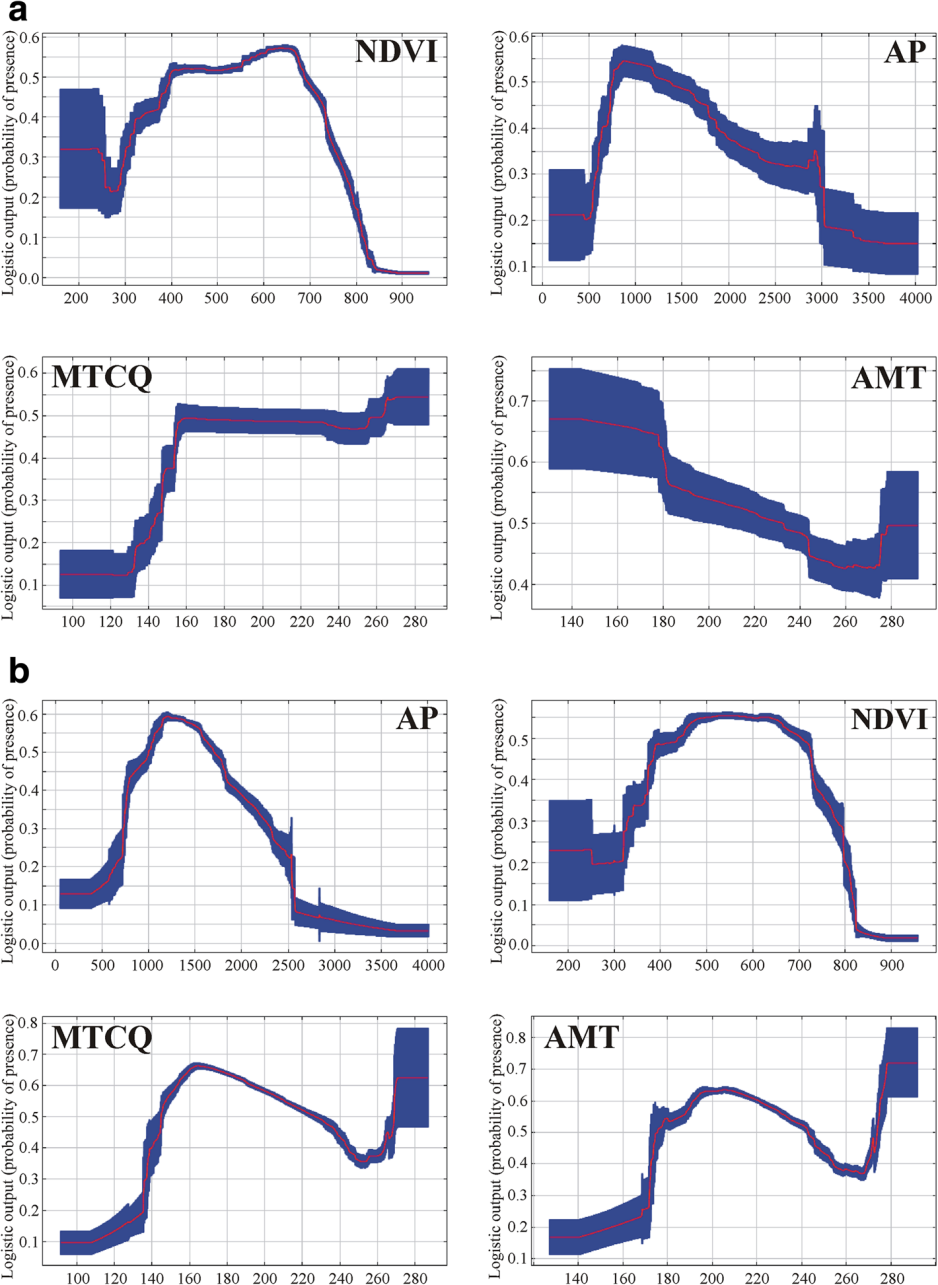
Response-curves of the variables. **a** American cutaneous leishmaniasis model (ACLM). **b** *Lutzomyia* (*N*.) *whitmani* model (LWM). Normalized difference vegetation index (NDVI), annual precipitation (AP, BIO12), mean temperature of coldest quarter (MTCQ, BIO11), annual mean temperature (AMT, BIO1). These curves show how each environmental variable affects the MaxEnt prediction when all environmental variables are used to build the model

**Fig. 3:**
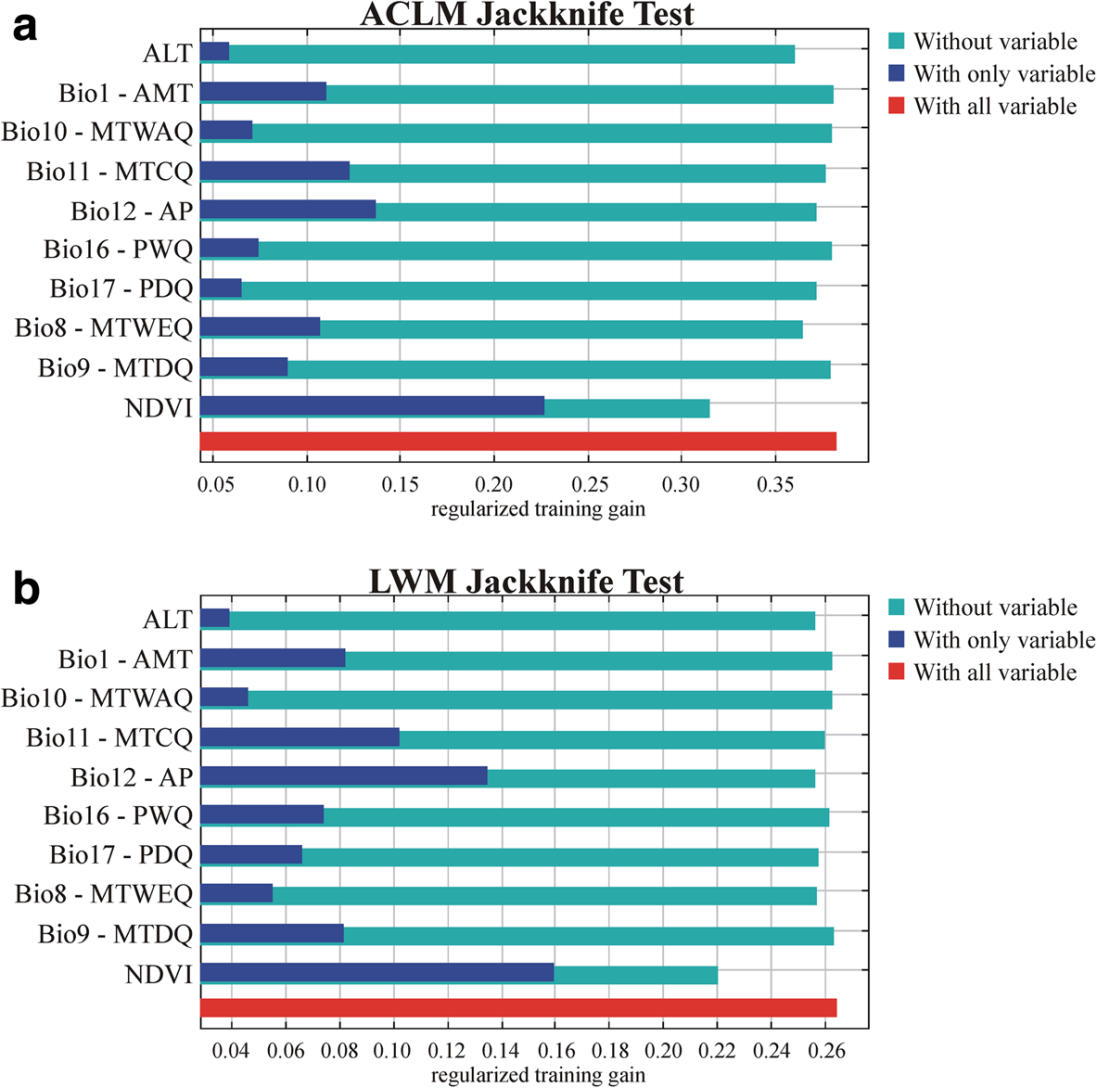
Jackknife test results of individual environmental variable importance in the development of the MaxEnt models. **a** American cutaneous leishmaniasis model (ACLM). **b** *Lutzomyia* (*N*.) *whitmani* model (LWM) jackknife test results. Red bars represent results for all environmental variables; dark blue bars represent results for each predictor variable alone, and light blue bars represent the drop in training gain when the variable is removed from the full model

The vegetation density index (NDVI) was the variable with the highest gain in the model when it was omitted or used alone, causing the significance of ACLM to decrease, respectively. The response curves for Environmental variables (EV) of this model indicate that ACL is strongly associated with intermediate density vegetation areas, zones with annual precipitation (AP) between 800–1200 mm, mean temperature of coldest quarter (MTCQ) above 16 °C, and annual mean temperature (AMT) lower than 23 °C (suitability of occurrence > 0.5) (Figs. 2a and 3a).

*Lutzomyia* (*N*.) *whitmani* was identified by LWM as a species that “preferentially occurs” in regions with relative moderate rainfall (AP between 1000–1600 mm), intermediate NDVI, and regions with MTCQ between 15–22 °C and AMT between 19–24 °C (Figs. 2b and 3a).

**Fig. 4:**
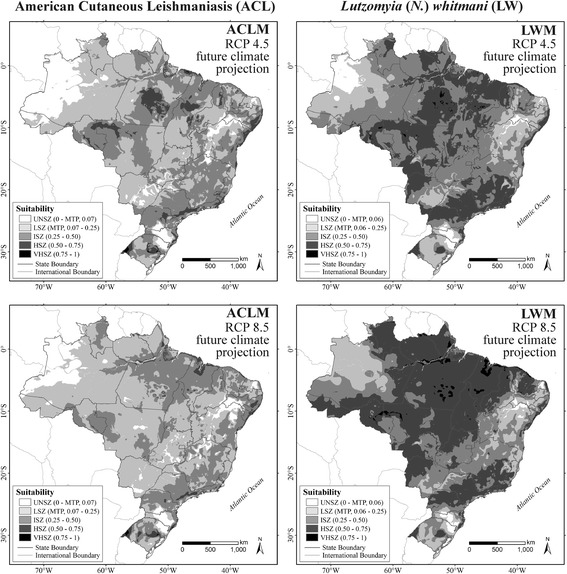
Future climate projections for American cutaneous leishmaniasis (ACL) and *Lutzomyia* (*N*.) *whitmani* (LW). Unsuitable zone (UNSZ; value pixel suitability < minimum training presence, MTP), low suitability zone (LSZ, value pixel suitability between MTP value and 0.25), intermediate suitability zone (ISZ, value pixel suitability between 0.25–0.50), high suitability zone (HSZ, value pixel suitability between 0.50–0.75), and a very high suitability zone (VHSZ, value pixel suitability > 0.75) identified

Figure 4 shows the future predicted distributions for ACL and *L.* (*N*.) *whitmani* in 2050, under both RCP 4.5 and RCP 8.5 (HadGEM2-ES model) for future climate scenarios. For ACL, these two projections differ moderately from current scenario (Fuzzy of 0.58 and 0.59 for RCP 4.5 and RCP 8.5, respectively) and are very similar to each other (Fuzzy of 0.75). Similar results were found in the projections for *L.* (*N*.) *whitmani* (Fig. 4), but with greater similarity (Fuzzy of 0.74 and 0.64, for current model *versus* RCP 4.5 and RCP 8.5, respectively, and Fuzzy of 0.77 between future climate scenarios).

Comparisons between the models for ACL and *L.* (*N*.) *whitmani* indicate high similarity. Fuzzy of 0.77 between current models, and 0.77 and 0.78 for RCP 4.5 and RCP 8.5 scenarios, respectively.

All the projections indicate expansion of the *L.* (*N*.) *whitmani* occurrence areas in the Brazilian territory. It increases by 5% in RCP 4.5 scenario and 7.6% in RCP 8.5 scenario. For ACL, the area gain values were higher (12.3% and 15.5% area gain for RCP 4.5 and RCP 8.5, respectively).

Suitable areas (above MTP cutoff values) for *L.* (*N*.) *whitmani* are more extensive than those suitable for ACL. Such areas cover 7,113,644.7 km^2^ of Brazilian territory, 1.2% more than the suitability for ACL (7,025,688.6 km^2^). In future projections, this behaviour is repeated, but with higher gain values in the suitable area for this vector (8.8% and 9.1% for RCP 4.5 and RCP 8.5, respectively).

**Fig. 5:**
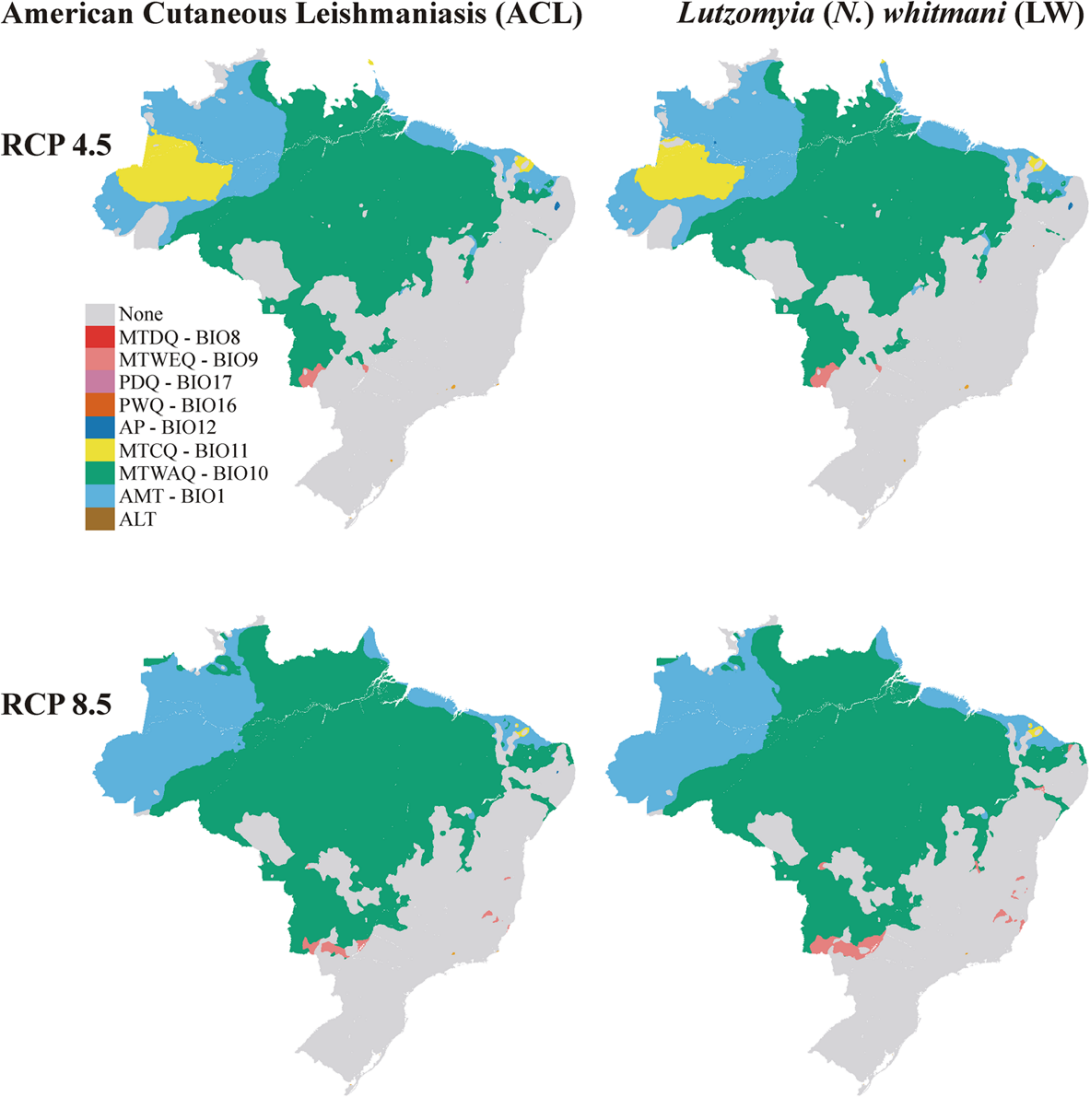
The most dissimilar variables (MoD) between current and future climate scenarios. For ACL and *L.* (*N*.) *whitmani* the mean temperature of warmest quarter (MTWAQ), mean temperature of coldest quarter (MTCQ) and annual mean temperature (AMT) were the drivers of current/future dissimilarity. Acronyms for environmental variables are provided in Table 1

Figure 5 shows the most dissimilar variables (MoD) between current and future climate scenarios. The MoD for a point *P* is the variable concerning which *P* has the smallest value of similarity, i.e. the variable driving the dissimilarity result [31]. For ACL and *L.* (*N*.) *whitmani*, the mean temperature of warmest quarter (MTWAQ), mean temperature of coldest quarter (MTCQ) and annual mean temperature (AMT) were the drivers of current/future dissimilarity.

## Discussion

The models generated for *L.* (*N*.) *whitmani* and ACL indicated a satisfactory predictive capacity. Future projections of LWM indicated a larger area of climatic suitability expansion for *L.* (*N*.) *whitmani* for northern Brazil and reinforces the expansion trend towards the South, as described by Peterson & Shaw [22].

*Lutzomyia* (*N*.) *whitmani* can “adapt” to environmental changes, new ecological niches, tolerating and overcoming the effects of changes that constantly occur in natural environments [22, 32]. According to Peterson & Shaw [22], *L.* (*N*.) *whitmani*, *L.* (*N*.) *intermedia* and *L. migonei*, phlebotomine vectors of ACL widely distributed in South America, by 2050 will have their climatic suitability areas increased. These species are expanding to different areas of the continent, with Peterson & Shaw [22], identifying the southern direction as the most evident for *L.* (*N*.) *whitmani*, replacing areas previously occupied by *L.* (*N*.) *intermedia* (*s.l.*) [22]. Our results corroborate that study. However, when we add 12 years to the *L.* (*N*.) *whitmani* occurrence records used by Peterson & Shaw [22] (a dataset before 2001), our current model shows the expansion predicted therein.

McIntyre et al. [33] contradicted Peterson & Saw [22], stating that *L.* (*N*.) *whitmani* overlaps with *L.* (*N*.) *intermedia* only in southeastern Brazil. In fact, our models indicate that *L.* (*N*.) *whitmani* can replace *L.* (*N*.) *intermedia* in the southeastern region of Brazil, participating in ACL transmission cycle, mainly in the states of Espírito Santo, Minas Gerais, Rio de Janeiro, and São Paulo, sharing in the transmission of *L.* (*V.*) *braziliensis*, as previously described by [34, 35]. As such, the expansion trend toward the south presented by our model represents the current distribution of *Lutzomyia* (*Nyssomyia*) *neivai*.

Other vectors of ACL present projections of future displacements towards higher latitudes, as observed in sand flies from Central and North America [36, 37]. *Phlebotomus ariasi* showed increased abundance at higher latitudes in central Spain. According to Gálvez et al. [38], the species would be migrating to these areas to compensate for the increase in temperatures in the region. Carvalho et al. [39] describe an expansion of *Lutzomyia* (*Nyssomyia*) *flaviscutellata* to the south and southeast of Brazil in the face of future climatic scenarios. Therefore, one can infer that the area of overlap between *L.* (*N*.) *flaviscutellata* and *L.* (*N*.) *whitmani* will be larger and more evident in the future.

The results point to the predicted expansion of *L.* (*N*.) *whitmani* in the northern region, especially the State of Amazonas: although future projections show that the Amazon region will become drier, as a consequence of the increase in intensity and duration of the dry season [40], *L.* (*N*.) *whitmani* remains present in the region and will have a more extensive climatic suitability area in the future. Considering the extensive latitudinal range of Brazil, regional climates play an important role in the definition of species distribution. According to Carvalho et al. [39], most projections of climate change endorse that vectors of diseases will find good climatic conditions for their geographical expansion in the higher latitudes during the coming decades.

In relation to the epidemiology of ACL in Brazil, the disease expansion process is related to environmental changes with new human cases being registered in areas of recent deforestation, mining, hydroelectric plant construction and population settlements [9, 12]. These changes in the transmission pattern favour the dispersion of wild animals and sand flies mainly to the peridomestic environment, where new transmission cycles can be established close to houses [9]. In this case, *L.* (*N*.) *whitmani* and *L.* (*N*.) *flaviscutellata* would be particularly good examples of species, in different epidemiological situations [31]. This relationship is identified in ACLM by the strong relation of the most suitable areas for ACL with areas of intermediate vegetation cover density. Therefore, the most conserved Amazonian areas are identified as unsuitable.

Future projections for ACL indicate an expansion to northwestern Brazil. This is more evident in RCP 8.5, which is more pessimistic in relation to policies to control the emission of greenhouse gases, adding 15.5% to the total area of occurrence of the disease. The lack of future scenarios of the change in density and vegetation cover, in the way of those that exist for climatic data, made it impossible to quantify the role of changes in forest cover in future forecasts. However, the known and progressive environmental degradation, associated with future climate predictions that indicate that the Amazon region will tend to become more suitable climatically for both ACL and *L.* (*N*.) *whitmani*, design a scenario of higher risk of cases of disease [39, 41].

The larger distribution predicted in the models for *L.* (*N*.) *whitmani* regarding ACL epidemiology is possibly related to the sole presence of the vector not being deterministic for the disease. Other factors influence pathogen transmission as well as the development of the disease. The ecoepidemiology of Brazilian ACL is a complex of epidemiological chains involving different parasites, vectors, and reservoirs. The transmission of the seven *Leishmania* spp., associated with ACL in Brazil involves different phlebotomine species that are closely associated with the parasite’s mammalian reservoirs (which range from xenathra to rodents to primates), resulting in a variety of transmission cycles in the different geographical regions in the country. However, the little difference between the areas identified as adequate for *L.* (*N*.) *whitmani* and ACL, associated with the high similarities between the models reinforce the geographical importance of this vector in the transmission of ACL.

## Conclusions

The models showed that continuous process of environmental degradation favours the establishment of *L.* (*N*.) *whitmani* and the occurrence of ACL. Future projections of ACL models indicate the ongoing process of disease expansion in the face of the predicted climatic changes and reinforce the broad geographical expanse of the disease. In this view and associated with the new epidemiological patterns resulting from the drastic environmental changes (coupled with the presence of highly adapted vectors, reservoirs, and parasites) the epidemiological scenario for ACL indicates a continuous increasing of human cases. The results presented here are expected to improve assessment of vector surveillance actions, consequently contributing to the promotion of health in risk areas for ACL associated to *L.* (*N*.) *whitmani*, projected for future scenarios in Brazil.

## Additional files

Additional file 1: **Table S1.** Compiled presence records of American cutaneus leishmaniasis (ACL). (XLSX 113 kb)

Additional file 2: **Table S2.** Compiled presence records of *Lutzomyia* (*N.*) *whitmani*. (XLSX 60 kb)

## Abbreviations

ACL: American cutaneous leishmaniasis
ACLM: American cutaneous leishmaniasis model
AMT: annual mean temperature
AP: annual precipitation
AR5: Fifth assessment report of the 2014 Intergovernmental Panel on Climate Change
ENM: ecological niche modelling
EV: environmental variables
HSM: habitat suitability map
IPCC: Intergovernmental Panel on Climate Change
LWM: *Lutzomyia* (*N*.) *whitmani* model
MaxEnt: Maximun entropy
MoD: most dissimilar variable
MTCQ: mean temperature of coldest quarter
MTP: minimum training presence
NDVI: Normalized Difference Vegetation Index
RCP: representative concentration pathway
